# Mental health and meaning in modern life among young adults in Norway

**DOI:** 10.1007/s44192-025-00317-z

**Published:** 2025-11-21

**Authors:** Lars Mandelkow, Odd Kenneth Hillesund

**Affiliations:** 1 Department of Psychology, Ansgar University College, Kristiansand, Norway; 2 Department of Psychosocial Health, University of Agder, Kristiansand, Norway

**Keywords:** Mental health, Young adults, Meaning in life, Social engagement, Screen time, Norway

## Abstract

Mental health concerns are increasing among young adults in Western societies, including Norway. While rising distress is well documented, fewer studies investigate how meaning in life and everyday social behaviour relate to mental well-being – especially in highly secular, individualised contexts. This study examines how mental health symptoms among Norwegian young adults are associated with perceived meaning, social commitment, screen time, and time spent in nature. A total of 713 first-year students aged 18–21 from different education institutions completed a survey including the SCL-10 (mental health symptoms), The Meaning and Purpose Scales (MAPS), and behavioural measures. Analyses included correlations, t-tests, MANOVA, and hierarchical regressions. Higher levels of psychological distress were strongly associated with lower perceived meaningfulness and higher crisis of meaning. Social commitment correlated with better mental health and stronger meaning scores across several MAPS domains, including spirituality and community connection. Screen time was positively associated with distress, while time in nature was linked to higher meaning. Guided by different theoretical psychological and sociological perspectives, the findings suggest that existential resources – especially meaningfulness, social engagement, and resonance with nature – play a central role in young people’s mental health. Conversely, excessive digital engagement may disrupt these sources of orientation. The study contributes to existential health research by combining psychological and sociocultural theory in a secular setting. Policy and educational strategies should support young people’s access to resonant, meaning-oriented environments, with special attention to screen balance, social participation, and nature-based experiences.

## Background

Mental health challenges are on the rise among young adults globally, with similar patterns observed in Norway. While these trends are well documented [1], less attention has been given to how young people’s sense of meaning in life relates to their mental health – particularly within the context of a highly secular, individualistic society. This study addresses this gap by exploring the relationship between mental health symptoms and meaningfulness, alongside social-interactional variables such as screen time, time spent in nature, and social commitment. Norwegian young adults provide a compelling case study of how meaning and mental health intersect in a highly secular context. While Norwegian culture has distinctive features compared to other Scandinavian societies, requiring some caution in generalisation [2], it illustrates broader Western trends of youth navigating modern pressures without strong religious frameworks.

### Young Norwegians’ mental health

Mental health challenges among young adults have become increasingly prominent in recent years, with anxiety, depression, and stress on the rise across Western societies. The transition to adulthood – marked by increased independence and decision-making – can be particularly stressful [3]. A recent systematic review reported that nearly one in five young Europeans suffer from a mental disorder, with a pooled prevalence of 15.5% [4]. The COVID-19 pandemic has further exacerbated this trend, doubling mental health issues among youth in the WHO European Region and contributing to rising suicide rates [5].

Factors linked to this development include increased digital engagement and exposure to social media [6], global and ecological anxiety [7], and changing societal norms. However, the narrative of a universal increase in mental distress remains contested; a Dutch cohort study found no significant rise in mental health problems among 19–24-year-olds from 2007 to 2017 [8].

In the Norwegian context, data from Potrebny, Nilsen [1] and Krokstad, Weiss et al. [9] show a clear increase in mental health symptoms among youth, particularly females. National “Ungdata” surveys confirm that while most adolescents report overall life satisfaction and strong relationships, issues like loneliness, stress, and reduced engagement in organized activities remain prevalent [10]. Notably, the combination of high digital activity and low social participation correlates with poorer mental health outcomes [11], underscoring the importance of balancing screen use with social and physical engagement [12, 13].

These findings suggest a paradox: young people may be “doing better, but feeling worse” [14], – doing better on global indicators such as overall life satisfaction, yet feeling worse when it comes to domain-specific symptom loads like stress, loneliness, and reduced participation. This paradox underscores the need to examine deeper existential dimensions of well-being, including meaning in life.

### Meaning in modern life

Norwegian society balances strong individual freedoms with collective responsibility [15]. Young Norwegians grow up in one of the world’s most egalitarian and affluent nations, yet they navigate an increasingly complex landscape marked by societal expectations, self-realization, and digital connectivity. These factors are likely to shape how young people experience meaning in life and may, in turn, influence their mental health.

Moreover, Norway provides a particularly relevant context because of its high level of secularization. Traditional religious institutions play a limited role in most young people’s lives, yet studies consistently show that religiosity and spirituality are protective factors for mental health in many societies, also for young people [16, 17]. In secular contexts, these functions are often fulfilled through alternative sources of meaning, such as community participation, creative pursuits, or personal growth. Scholars of implicit religion argue that frameworks once grounded in religion may re-emerge in secular forms of ritual, values, and belonging [18, 19]. This makes the Norwegian case important for exploring how existential resources are mobilized outside formal religion, and how their absence may exacerbate crises of meaning.

The psychology of religion and existential psychology have long highlighted the central role of meaning in human well-being. Frankl’s classic work Man’s Search for Meaning [20] argued that finding purpose is essential for resilience, especially under adversity. Building on this foundation, Schnell’s theory of meaning in life offers a comprehensive framework for understanding how individuals derive meaning through sources such as self-development, relatedness, and prosocial engagement [21, 22]. The sense of meaning in life can be defined as the extent to which individuals perceive their lives as significant, purposeful, and coherent [23]. Schnell’s framework operationalises this concept through the dual dimensions of meaningfulness and crisis of meaning, which capture the presence of purpose and the struggle with its absence.

In a society that places high value on personal growth, self-realization, and autonomy [24], *self-oriented meaning* plays a particularly strong role. Many young Norwegians derive meaning from pursuing education, engaging in creative expression, and striving for independence [25]. For instance, initiatives like Norway’s folk high schools provide students with opportunities to explore values and interests in non-competitive, reflective settings [26]. However, the effects of such environments on existential and psychological well-being remain under-researched.

In accordance with earlier Norwegian studies [27], Schnell also highlights the importance of *relatedness and community* as key meaning sources. Norwegian adolescents often participate in organized activities such as sports and cultural groups but increasingly turn to social media for connection and identity affirmation. While these digital spaces can foster a sense of community, they also expose youth to pressures that may undermine meaningful relationships and self-worth [28].

When these meaning structures are disrupted – through loneliness, societal pressure, or digital overload – young people may experience *existential vacuum* – a state of emptiness that arises when the will to meaning is frustrated [20]. While this notion refers to a general absence of purpose, Schnell’s concept *of crisis of meaning* captures a more dynamic psychological state in which individuals actively grapple with the loss or questioning of meaning. Such conditions can compromise resilience and are increasingly reflected in mental health concerns and social disconnection among Norwegian youth, suggesting that traditional meaning sources like family and community engagement may be weakening [10, 29].

In this article, Schnell’s theory of meaning in life serves as the primary framework. Rosa’s resonance theory and Antonovsky’s sense of coherence are introduced as complementary perspectives, enriching but not competing with Schnell’s model by situating meaning within sociocultural and health-related dynamics.

From a sociological point of view, Rosa [30, 31] critiques modern societies for fostering alienation through relentless acceleration and the prioritisation of control and efficiency. As an alternative, he introduces resonance – responsive, transformative connection between individuals and the world (e.g., through relationships, art, or nature), distinct from passive or instrumental contact – that enable individuals to experience meaning. This perspective enriches Schnell’s framework by showing how access to meaning is shaped not only by individual orientations but also by institutional and societal conditions. The connection between meaning and health has deep roots in the salutogenic tradition [32] which emphasized the motivational power of perceiving life as coherent and meaningful [33]. Antonovsky’s model, originated in medical sociology, provided a foundation for subsequent existential approaches such as Schnell’s multidimensional theory of meaning, which extends the salutogenic idea by differentiating between coherence, significance, and belonging.

These perspectives together situate meaning at the intersection of psychology, sociology, and health research. While the psychology of religion has underlined the protective role of spirituality and transcendence, secular contexts like Norway highlight how existential resources are increasingly mobilised in non-religious forms, underscoring the value of integrating psychological and sociological perspectives.

Moreover, meaning in life develops dynamically across the life span: while the presence of meaning generally increases with age, the search for meaning peaks in adolescence and young adulthood, making this period especially sensitive for existential development [34, 35]. Social connectedness and commitment are consistently associated with higher levels of meaning [36]. Earlier sociological work has also highlighted how meaning in life can be understood as a relational and cultural construct [37], a perspective that remains underrepresented but enriches psychological approaches. These findings situate the present study within a broader literature while underscoring the need for research in highly secular contexts like Norway, where traditional meaning sources may be less available.

Although prior research suggests that meaning in life can be protective against mental health issues in adolescence [38, 39], empirical studies linking meaning, mental health, and contextual factors such as screen time and nature exposure remain scarce. In secular contexts, this gap is especially relevant, as traditional sources of transcendence and belonging may be replaced by more individualized or mediated experiences. Sociodemographic factors also play a role, influencing both meaning in life and its relation to mental health [38, 40]. In Norway, folk high school students – who make up most of our sample – are broadly comparable to university students but not fully representative of the youth population [41]. Taken together, these contextual and demographic considerations underline the need for research that integrates existential and behavioural perspectives when examining youth well-being.

This study addresses this gap by examining how mental health correlates with perceived meaning in life and social-interactional variables among young adults in Norway. In doing so, it contributes to a broader understanding of existential health in a society navigating the tensions between autonomy, connectivity, and well-being.

### Research questions

1. How does the mental health of young adults in Norway, as measured by the SCL-10, correlate with various sources of meaning and purpose in life, as assessed by the MAPS instrument?
2. To what extent is social commitment associated with mental health and perceived meaning in life among Norwegian young adults?
3. How do screen time and time spent in nature relate to mental health and sense of meaning in life in this population?

## Method

### Participants and recruitment

Participants were recruited using two strategies. First, all students enrolled in Norwegian folk high schools (folkehøgskoler) – non-formal post-secondary boarding institutions focusing on personal development attended by 12% of Norwegians after school – received a digital invitation via their school’s email distribution system. The invitation included a link to the online survey and was distributed in collaboration with the national Folk High School Council. Second, first-year students at a Norwegian university and university college were invited to participate either during class or via learning platforms. The university sample included students in psychology, music, education, nursing, and engineering.

For the present analyses, we included only participants aged 18 to 21 years who were in their first year of study and had not previously completed military service, vocational education, or another folk high school year. This resulted in a final sample of 713 participants (63% female), with 541 from folk high schools and 172 from higher education institutions. Given that the survey was conducted during the first days of the academic year – before sustained exposure to institutional culture – we treated participants as one analytic group. This choice was further supported by preliminary analyses showing no significant baseline differences in mental health symptom scores across institution types.

### Ethics

Data were collected using Nettskjema (University of Oslo), an online survey tool designed to comply with Norwegian privacy requirements. Participation was voluntary and anonymous: information about the study’s purpose, voluntary nature, and data protection was provided both in the invitation and on the survey’s introductory page, and participants indicated consent by clicking “Participate.” No identifying information was collected. After ethical assessment by the head of research at Ansgar University College and the Norwegian Folk High School Council, the study protocol was approved by the Norwegian Agency for Shared Services in Education and Research (ref. no. 853306).

### Measures

The survey started with some demographic questions (age group, gender, university/folk high school, migration background) and the following behavioural questions:

#### Social commitment

Social commitment was measured with the question: “How often do you participate in group activities outside school (e.g., choir, sports, volunteer work)?” Responses were given on a 5-point Likert scale from never (1) to several times per week (5). For the analysis, participants were categorized into three levels of social commitment. These groups were created directly from the predefined response categories, not through statistical cut-offs: low (never or once a month), moderate (several times a month or once a week), and high (several times a week).

#### Screen time

Participants reported their average daily time spent on social media (excluding music streaming) via a numeric entry field (in hours). This variable was used as a proxy for digital engagement, with limitations noted in the discussion.

#### Time spent in nature

Participants were asked: “How often do you spend time in nature (e.g., hiking, walking, outdoor recreation)?” Responses were recorded on a 4-point scale ranging from rarely (1) to daily (4). For the analysis, participants were categorized into three levels of time spent in nature (response-driven, see above): low (rarely), moderate (up to several times a week), and high (daily).

#### Video gaming

Regular video gaming was measured dichotomously (yes/no) with the question: “Do you play video games regularly (at least once a week)?”

#### SCL-10

The SCL-10 (Symptom Checklist-10) is a shortened version of the Symptom Checklist-90 (SCL-90), designed to screen for general psychological distress. The Norwegian version of the SCL-10 is a validated tool used for measuring symptoms related to mental health, including anxiety and depression [43]. It includes 10 items that ask respondents to rate how much they have experienced specific psychological and physical symptoms over the past week, using a 4-point Likert scale ranging from “not at all” (1) to “very much” (4). Example items include: “During the past week, … how much have you felt tense or keyed up?”, “… how often have you felt hopeless about the future?”, and “… how much have you experienced difficulty in sleeping (trouble falling asleep, waking up during the night)?”

The Norwegian SCL-10 is commonly used in research and clinical settings due to its brevity and reliability in assessing general psychological distress levels. It covers core symptoms such as feelings of nervousness, restlessness, low energy, and hopelessness. The survey is often applied as a quick assessment tool to identify individuals who may require further psychological evaluation or intervention (a common cut-off sum score is a mean of 1,85). The SCL-10 has been adapted and tested to ensure cultural relevance and linguistic accuracy in the Norwegian context, making it a valuable tool for mental health research and practice in Norway [43].

#### MAPS

The Meaning and Purpose Scale (MAPS), developed by Schnell and Danbolt [44], is an instrument that originated from Schnell’s work on the Sources of Meaning and Meaning in Life Questionnaire (SoMe) [45]. The SoMe was initially created to explore various sources of meaning and their significance in individuals’ lives. Over time, Schnell refined this concept into the MAPS survey, which continues to build on this foundation. MAPS is a comprehensive tool designed to measure various aspects of meaning in life and related constructs and includes specific scales that assess different dimensions of personal meaning and engagement. In this study, respondents rated its 23 items (in a Norwegian version) on a 5-point Likert scale from 0 (strongly disagree) to 4 (strongly agree), due to a programming oversight (the usual MAPS scaling is 6-point; reliability remained high, confirming data quality). The subscales explore the *sense of meaningfulness* (Coefficient α = 0.867; example item: “I have found my path in life.”), *crisis of meaning* (Coefficient α = 0.850; example item: “My life feels empty.“), *spirituality* (Coefficient α = 0.964; example item: “My daily actions are determined by my belief in God/a higher power.“), *sustainability* (Coefficient α = 0.763; example item: “I feel a strong connection with all living beings on Earth.“), *personal growth* (Coefficient α = 0.823; example item: “I set goals in life to keep learning”), *adherence to values* (Coefficient α = 0.636; example item: “I always follow laws and regulations”), and *community* (Coefficient α = 0.734; example item: “Closeness to others is the foundation of a good life.“). The Norwegian MAPS survey is employed in various research and educational contexts to understand how different aspects of meaning contribute to personal and social well-being.

### Hypotheses and analysis

To answer the research questions in this study, a series of statistical analyses were conducted to explore the relationships between mental health, sources of purpose and meaning, and other relevant variables among young adults in Norway. To conduct a preliminary exploration of the sample, a one-way ANOVA was used to examine differences in mental health scores across gender groups, while a t-test was performed to assess differences in mental health scores between participants with and without a migration background.

*Research Question 1: How does the mental health of young adults in Norway*,* as measured by the SCL-10*,* correlate with various sources of meaning and purpose in life*,* as assessed by the MAPS instrument?*

To address this question, the Pearson product-moment correlation coefficient was calculated to determine the strength of relationships between mental health (measured by the SCL-10 sum score) and scores from different subscales of the MAPS survey, which assess *sense of meaningfulness*, *crisis of meaning*, and various sources of meaning in life.

Based on prior research and theoretical frameworks, negative relationships were expected between the SCL-10 score (higher scores indicating poorer mental health) and most MAPS subscales, except for the *crisis of meaning* subscale, which was anticipated to show a positive correlation with mental health symptom severity. Since multiple correlation tests were conducted, alpha levels were adjusted using the Bonferroni method [46].

*Research Question 2: To what extent is social commitment associated with mental health and perceived meaning in life among Norwegian young adults?*

To investigate this question, a one-way Multivariate Analysis of Variance (MANOVA) was employed to compare mean SCL-10 and MAPS scores across groups categorized by levels of social commitment (low, medium, high). Post hoc analyses were conducted to identify specific differences between the groups. Additionally, multiple linear regression analysis was used to further explore the relationship by controlling for demographic variables such as age and migration background. Social commitment was expected to be positively correlated with both mental health (lower SCL-10 scores) and the experience of meaning (higher MAPS scores).

*Research Question 3: How do screen time and time spent in nature relate to mental health and sense of meaning in life in this population?*

To answer this question, hierarchical regression analyses were conducted in two steps. In the first step, demographic control variables (gender and migration background) were entered. In the second step, behavioural variables (time spent online and time spent in nature) were added as predictors. The dependent variables were mental health scores (SCL-10) and the MAPS score measuring the sense of meaning in life. It was hypothesized that increased screen time would negatively impact mental health (higher SCL-scores) and meaning (lower MAPS scores), whereas more time spent in nature would positively influence these outcomes.

All statistical analyses were conducted using SPSS version 29.

## Results

### Sample

The analytic sample consisted of 713 participants aged 18 to 21. Of these, 172 (24%) were first-year university or university college students, while the remaining 541 (76%) attended one of 83 Norwegian folk high schools. The gender distribution was 63% female, 31% male, and 6% identifying as “other” or choosing not to disclose. A migration background was reported by 6% of participants (see Table 1).

**Table 1:** Sample characteristics, n = 713, age 18–21 years

	Total		Folk high school		University/college		
	*n*	%	*n*	%	*n*	%	*p*-value difference FHS/Uni
Participants	**713**	***100***	541	*75.9*	172	*24.1*	
Response rate				*26*		*17*	
Gender							*p* < .001
Female	**447**	***62.7***	317	*58.9*	130	*75.6*	
Male	**224**	***31.4***	186	*34.6*	38	*22.1*	
Other/ prefer not to answer	**39**	***5.5***	35	*6.5*	4	*2.3*	
Missing	**3**						
Background							*p* = .366
Migration	**42**	***5.9***	31	*5.8*	11	*6.4*	
No migration	**663**	***93***	502	*93.1*	161	*93.6*	
Prefer not to answer	**6**	***0.8***	6	*1.1*	0	*0.0*	
Missing	**2**						

## Mental health by gender and migration background

The SCL-10 sum score, indicating poor mental health, differed significantly across gender groups according to an ANOVA analysis, F(2, 695) = 26.39, *p* < .001, η² = 0.071, indicating a moderate effect size. Mean scores were lowest among males (M = 1.66, SD = 0.64), higher among females (M = 1.87, SD = 0.65), and highest among participants identifying as “other/prefer not to answer” (M = 2.45, SD = 0.59). Levene’s test indicated homogeneity of variances (*p* = .538). Tukey post hoc comparisons revealed significant differences between all gender groups (*p* < .001). No significant differences in SCL-10 scores were found between participants with and without a migration background.

**Fig. 1:**
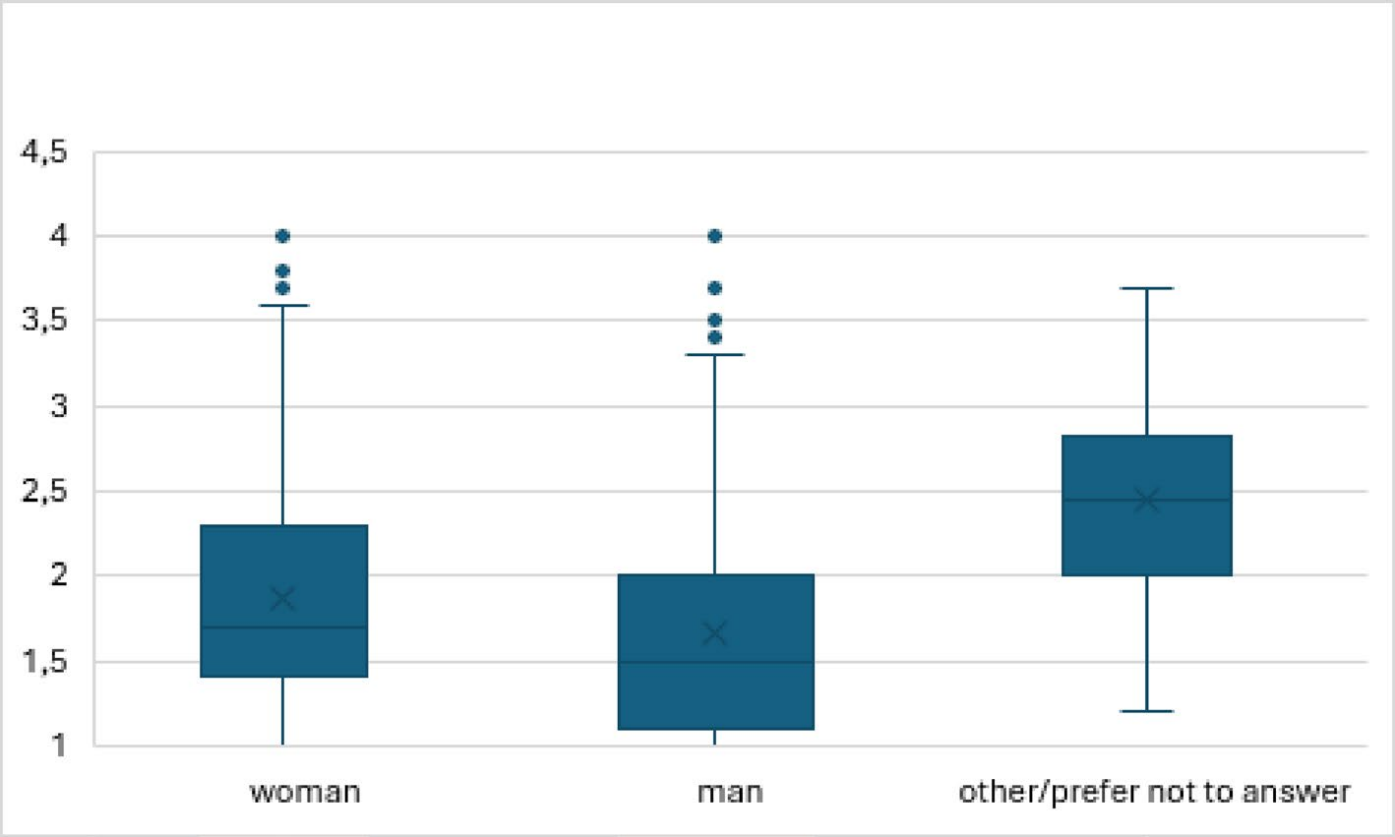
Mental health (SCL-10 mean) distribution in the different gender groups

### Research question 1: mental health and meaning

The association between mental health symptoms (SCL-10) and sources of meaning (MAPS subscales) was assessed using Pearson product–moment correlations. Assumptions of normality, linearity, and homoscedasticity were met, and significance levels were Bonferroni-adjusted for multiple comparisons.

A strong negative correlation was found between mental health and sense of meaningfulness (*r* = –.562, *p* < .001), indicating that higher psychological distress was associated with lower perceived meaning in life. Conversely, a strong positive correlation was observed between mental health symptoms and crisis of meaning (*r* = .587, *p* < .001), suggesting that higher symptom severity coincided with a pronounced existential crisis.

Among the MAPS purpose factors, community connection demonstrated a moderate negative correlation with mental health symptoms (*r* = –.247, *p* < .001). Smaller but statistically significant negative correlations were found for spirituality (*r* = –.165, *p* < .001) and personal growth (*r* = –.147, *p* < .001), indicating that higher distress was associated with lower importance of faith and growth. No significant correlations were found for adherence to values or sustainability after Bonferroni adjustment (see Table 2).

**Table 2:** Correlations between mental health (SCL-10) and meaning (MAPS scores), Bonferroni-adjusted

Pearson Product-Moment Correlations							
Sumscore mental health	Meaning-fulness	Crisis of meaning	Spirituality	Personal growth	Adherence to values	Sustainability	Community
Pearson Correlation	− 0.562^**^	0.587^**^	− 0.165^**^	− 0.147^**^	− 0.077	− 0.063	− 0.247^**^
Sig. (2-tailed), Bonferroni-adjusted	< 0.001	< 0.001	< 0.001	< 0.001	0.308	0.679	< 0.001
N	692	677	695	687	686	687	691

** Correlation is significant at the < 0.001 level (2-tailed).

### Research question 2: social commitment, mental health, and meaning

To assess the impact of social commitment on mental health and meaning, participants were divided into three groups (low, moderate, and high) based on self-reported frequency of participation in group activities outside school. Although Shapiro–Wilk tests indicated non-normal distributions for some dependent variables, MANOVA was conducted, as the procedure is considered robust to moderate violations of normality in large samples [47]. Levene’s test showed violations of homogeneity of variance for several MAPS variables; however, the analysis proceeded in line with recommendations by Ateş, Kaymaz et al. [48], who note that MANOVA is generally robust under these conditions.

The one-way MANOVA revealed a statistically significant effect of social commitment on the combined dependent variables, Wilks’ Λ = 0.874, F(16, 1262) = 5.475, *p* < .001, partial η² = 0.065, indicating a moderate multivariate effect.

Follow-up univariate ANOVAs demonstrated significant between-group differences for all outcome variables except *adherence to values* (see Table 3).

**Table 3:** Results of post-hoc univariate ANOVAs of social commitment, mental health, and meaning

Measure	df1	df2	F-value	*p*-value	Partial η²
SCL sum score	2	638	10.186	< 0.001	0.031
Sense of meaningfulness	2	638	22.354	< 0.001	0.065
Crisis of meaning	2	638	18.924	< 0.001	0.056
Spirituality	2	638	22.061	< 0.001	0.065
Personal growth	2	638	6.542	= 0.002	0.020
Adherence to values	2	638	0.395	= 0.674	0.001
Sustainability	2	638	6.620	= 0.001	0.020
Community	2	638	11.659	< 0.001	0.035

**Fig. 2:**
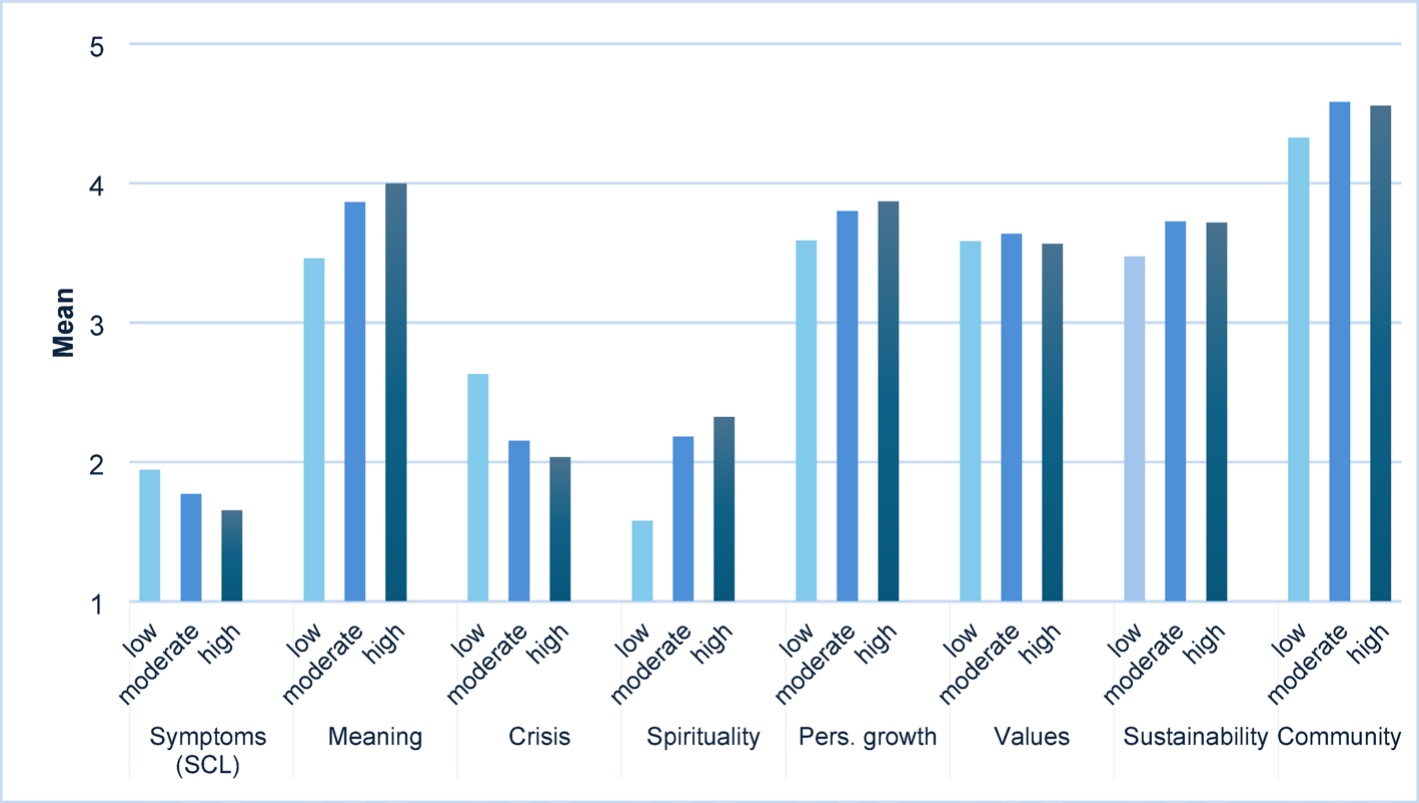
Comparisons of groups of social commitment on SCL and MAPS scores

Games–Howell post-hoc tests were used to account for unequal variances and sample sizes. Results indicated that both moderate and high social commitment groups differed significantly from the low social commitment group on most variables, with higher levels of social commitment associated with lower SCL-10 scores and higher levels of meaning across most MAPS domains (see Table 4; Fig. 2). No significant differences were found between the moderate and high groups.

**Table 4:** Multiple comparisons of groups of social commitment on SCL and MAPS scores

Measure	Compared groups of social commitment		M_Diff_	SEM	Sig.	95% confidence interval	
						Lower bound	Upper bound
SCL mean	Low	Moderate	0.1727*	0.06218	0.016	0.0263	0.3191
	Low	High	0.2916*	0.06725	0.000	0.1330	0.4502
	Moderate	High	0.1189	0.07565	0.260	− 0.0594	0.2972
Meaningfulness	Low	Moderate	− 0.4043*	0.08213	0.000	− 0.5976	− 0.2110
	Low	High	− 0.5392*	0.08769	0.000	− 0.7460	− 0.3325
	Moderate	High	− 0.1349	0.09681	0.345	− 0.3631	0.0932
Crisis of meaning	Low	Moderate	0.4795*	0.10126	0.000	0.2412	0.7178
	Low	High	0.5968*	0.11173	0.000	0.3333	0.8603
	Moderate	High	0.1173	0.12353	0.609	− 0.1738	0.4084
Spirituality	Low	Moderate	− 0.6041*	0.12901	0.000	− 0.9083	− 0.2998
	Low	High	− 0.7452*	0.14847	0.000	-1.0963	− 0.3940
	Moderate	High	− 0.1411	0.18105	0.716	− 0.5678	0.2856
Personal growth	Low	Moderate	− 0.2123*	0.07708	0.017	− 0.3937	− 0.0309
	Low	High	− 0.2796*	0.09093	0.007	− 0.4942	− 0.0650
	Moderate	High	− 0.0673	0.09875	0.774	− 0.3001	0.1655
Adherence to values	Low	Moderate	0.0562	0.08482	0.832	− 0.5678	0.2856
	Low	High	− 0.0354	0.09334	0.508	− 0.3631	0.0932
	Moderate	High	0.0088	0.06883	0.723	− 0.2145	0.2297
Sustainability	Low	Moderate	− 0.2504*	0.08064	0.006	− 0.4402	− 0.0606
	Low	High	− 0.2428*	0.08360	0.011	− 0.4399	− 0.0457
	Moderate	High	0.0076	0.09426	0.996	− 0.2145	0.2297
Community	Low	Moderate	− 0.2542*	0.05217	0.000	− 0.3769	− 0.1315
	Low	High	− 0.2289*	0.06833	0.003	− 0.3901	− 0.0678
	Moderate	High	0.0253	0.06741	0.925	− 0.1338	0.1843

*The mean difference is significant at the 0.05 level

These findings underscore the consistent association between frequent social engagement and both lower psychological distress and higher perceived meaning in life, particularly with regard to spirituality, personal growth, sustainability, and community.

### Research question 3: screen time, time spent in nature, meaning, and mental health

To examine how lifestyle-related factors predict mental health and meaning, two hierarchical multiple regressions were conducted using (1) mental health (SCL-10 mean score) and (2) sense of meaning in life (MAPS score) as dependent variables. Gender and migration background were entered in Step 1, followed by daily screen time and time spent in nature in Step 2.

The final model significantly predicted mental health, F(4, 687) = 10.89, *p* < .001, and sense of meaning in life, F(4, 688) = 10.03, *p* < .001. For both outcomes, the full model explained approximately 6% of the variance (adjusted R² = 0.05), indicating a small but meaningful effect.

In the model predicting poor mental health, only screen time emerged as a significant predictor (β = 0.237, *p* < .001), while gender, migration background, and time spent in nature were not significant. In contrast, in the model predicting meaning in life, all predictors except migration background were significant: gender (β = –0.113, *p* = .002), time spent in nature (β = 0.132, *p* < .001), and screen time (β = –0.122, *p* = .001), see Table 5.

Additionally, time spent in nature was negatively correlated with screen time, *r* = –.121, *p* < .001, and with SCL-10 mental health scores, *r* = –.080, *p* < .05. These correlations support the regression findings and suggest a small but consistent relationship between outdoor engagement and both well-being and meaning.

**Table 5:** Regression coefficients for the prediction of poor mental health and sense of meaning in life

Model	Predictor	Standardised coefficient	95% confidence interval		Sig.
			Lower bound	Upper bound	
(1) Poor mental health (SCL-10)	Gender	0.026	− 0.052	0.111	0.479
	Migration	− 0.034	− 0.279	0.102	0.361
	Time in nature	− 0.025	− 0.128	0.064	0.511
	**Screen time**	0.237	0.059	0.112	< 0.001
(2) Sense of meaning in life	**Gender**	− 0.113	− 0.286	− 0.062	0.002
	Migration	0.065	− 0.029	0.495	0.081
	**Time in nature**	0.132	0.106	0.370	< 0.001
	**Screen time**	− 0.122	− 0.097	− 0.024	0.001

**Bold text** indicates that the predictor is significant at the 0.05 level.

### Correlation between daily screen time and poor mental health (SCL-10)

**Fig. 3:**
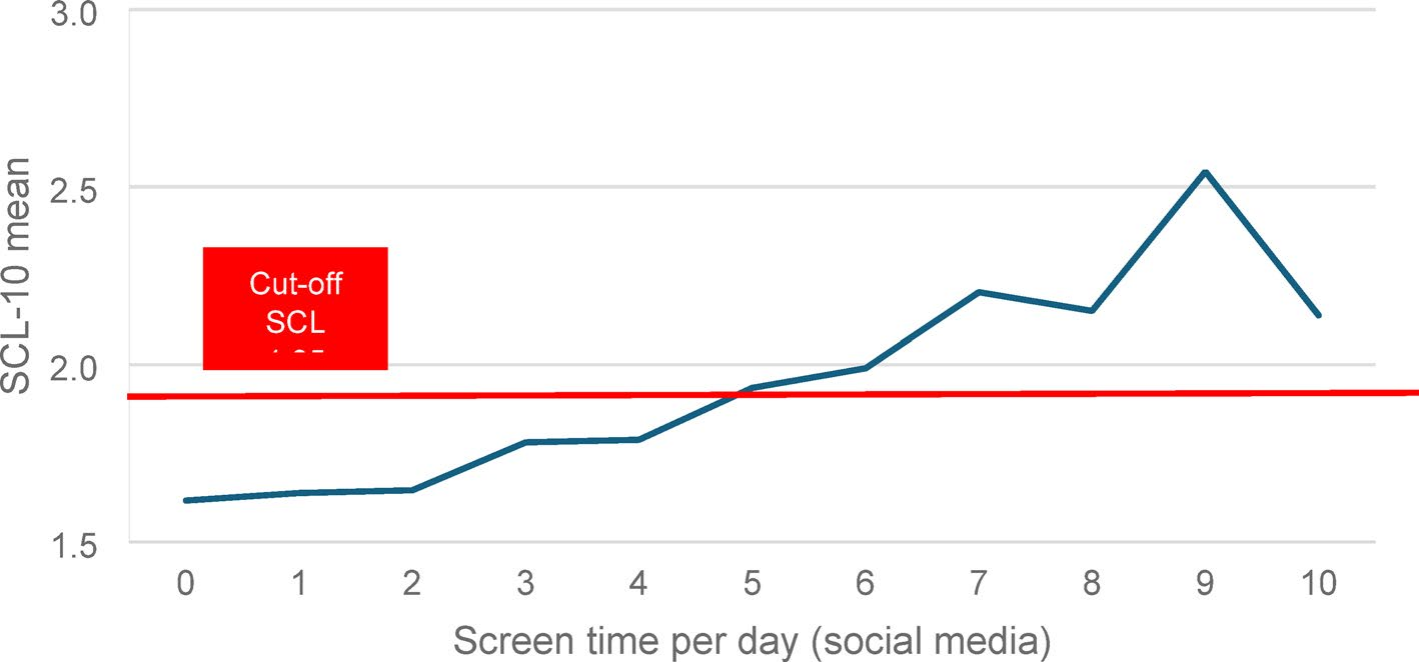
Correlation between daily screen time and poor mental health (SCL-10)

A further analysis revealed significant associations between daily screen time, regular video gaming, and poor mental health (see Table 6). Daily screen time showed the strongest correlation with SCL-10 scores, *r* = .239, *p* < .001 (see Fig. 3). While regular video gaming was negatively associated with mental health (*r* = –.128, *p* < .01) and screen time (*r* = –.137, *p* < .01), the effect size was small.

Among the behavioural predictors, screen time was the only variable consistently associated with both poorer mental health *and* reduced sense of meaning in life, highlighting its distinct role in shaping well-being (see Table 5; Fig. 3).

**Table 6:** Correlation between Poor Mental Health (SCL-10), Screen Time and Video Gaming

Variable	*N*	M	SD	1	2	3
(1) Mental health (SCL-10)	700	1.8381	0.66732	1	–	-
(2) Screen time	703	4.01	1.853	0.239**	1	-
(3) Video Gaming	710	1.69	0.462	− 0.128**	− 0.137**	1

**Correlation is significant at the 0.01 level (1-tailed)

## Discussion

This study investigated the relationships between mental health, meaning in life, and behavioural patterns among young adults in Norway. Three central findings emerged:

### Mental health and sources of meaning

Higher symptom scores on the SCL-10 were strongly associated with a lower sense of meaningfulness and community connection, while a crisis of meaning showed a strong positive correlation with symptom severity. This pattern underscores the pivotal role of meaning in psychological well-being.

### Social commitment

Participants with higher levels of social commitment reported better mental health and a stronger sense of meaning. These associations were particularly strong for the MAPS dimensions of meaningfulness and community, supporting the protective effect of regular social engagement.

### Screen time and time in nature

Daily screen time was positively associated with poorer mental health, while time spent in nature showed a small but significant positive correlation with a sense of meaning. These findings reflect the contrasting influences of digital and nature-based activities on youth well-being.

### Meaning and mental health

The findings from this study underscore the central role of meaning in life for young adults’ psychological well-being. A strong negative correlation between a sense of meaningfulness and symptoms of poor mental health (*r* = –.562, *p* < .001) supports Schnell’s [21] model, which conceptualizes meaning as a buffer against existential distress. Schnell’s theory posits that meaning arises from a constellation of sources – such as self-development, relationships, values, and spirituality – that together promote mental resilience. Conversely, the strong positive correlation between crisis of meaning and SCL-10 scores (*r* = .587, *p* < .001) suggests that psychological symptoms are closely linked to a loss of orientation or purpose.

While this study does not directly investigate the structural relationships between meaningfulness, crisis of meaning, and sources of meaning – as in earlier SoMe research [45], 2009) – the observed correlations indicate that these dimensions remain conceptually interrelated. For example, higher levels of community, spirituality, and personal growth were each associated with lower symptom levels, complementing the strong inverse correlation between meaningfulness and mental health, and the positive association between crisis of meaning and symptoms. Our findings suggest that promoting meaningful engagement – whether through community participation, creativity, or reflection – could mitigate such risks in emerging adults. However, while the strong correlation between crisis of meaning and psychological distress highlights their close interconnection, the cross-sectional design prevents conclusions about directionality. It is equally plausible that existential disorientation increases vulnerability to distress as that psychological symptoms erode one’s sense of purpose. Future longitudinal studies are needed to disentangle these pathways.

While only Schnell’s theory is directly operationalized here, the broader significance of our findings can be illuminated through complementary perspectives. For example, moderate negative correlations between mental health symptoms and MAPS subscales like community connection (*r* = –.247, *p* < .001) and spirituality (*r* = –.165, *p* < .001) illustrate how meaning is not only individual but also relational and transcendent. These findings resonate with Frankl’s assertion [20] that meaning – especially when grounded in relationships or spiritual experience – offers psychological anchoring during times of uncertainty or suffering.

Other frameworks highlight how commitment to meaningful activity – especially in collective or ritualised forms – can help individuals move beyond a purely achievement-focused lifestyle. Han [49] describes how rituals create distance from the self, de-psychologising and externalising inner experience. Such practices may foster intuitive, intersubjective presence and support mental regulation. La Cour and Hvidt [50] similarly argue that spirituality and connectedness – whether religious or secular – are integral to existential health. In the light of such interpretative extensions, arenas such as the Norwegian folk high schools [26] appear promising for fostering resonance [30], as daily interaction and shared practices enable reciprocal engagement and belonging. While more data are needed, their ethos of non-competitive, interest-driven learning corresponds to Schnell’s sources of meaning, particularly personal growth and community, by providing structured opportunities for exploration without performance pressure.

From the perspective of psychological coping, Antonovsky’s classical theory of *Sense of Coherence* (SOC) complements this interpretation by emphasising meaningfulness as the motivational core of human adaptation. Without meaningfulness, individuals are less likely to engage with life or perceive it as coherent and manageable [33]. Thus, the SOC model helps explain the observed link between existential orientation and mental health, particularly in contexts where coherence is challenged – such as the high-choice, high-pressure conditions of modern life.

Taken together, our findings and different sociological and psychological lenses reinforce the view that meaning in life is a multidimensional and context-sensitive construct – rooted in personal, social, and transcendent sources – and that associating it with mental health outcomes makes sense. Interventions aiming to support young adults’ well-being should consider how to promote varied and resonant sources of meaning, including relationships, nature, spirituality, and civic engagement. Further research should evaluate such initiatives, particularly in settings like folk high schools, to explore their potential for cultivating existential resilience.

### Suffering a modern life

The findings of this study reflect the growing complexities of modern life, where rising mental health diagnoses among young adults point to significant sociocultural and existential challenges. These trends can be contextualised by drawing on sociological and psychological theories, particularly those of Rosa [30], Brinkmann [51], and Madsen [52], who comment on Western societies and critique the ways in which contemporary Western societies shape experiences of distress, well-being, and identity.

Rosa argues that contemporary life fosters alienation across multiple domains, contrasting this with four dimensions of resonance: social (deep reciprocal relationships), material (engagement with nature and objects), existential (connection to art, spirituality, or history), and self-resonance (meaningful reflection on one’s inner life). Resonance, according to Rosa [30, 53, 54] is not a purely individual pursuit but emerges within institutional and societal frameworks. When institutions prioritize speed, control, and optimization over openness and relational depth, they hinder the possibility of resonance. Conversely, social structures that support unpredictability and genuine engagement foster well-being.

Although our measures did not directly operationalize Rosa’s concept of resonance, the observed associations (e.g., between community, spirituality, and better mental health) are consistent with his claim that “our relationship to the world” matters. By contrast, excessive screen time – linked here with poorer mental health – illustrates Rosa’s concept of digital alienation, where superficial interactions displace depth and reciprocity. These patterns highlight the importance of environments that facilitate resonance rather than acceleration.

Brinkmann and Madsen add further sociocultural critique that can contextualise the correlations between MAPS results and reported mental health symptoms. Both argue that therapeutic culture reframes life struggles as individual psychological problems. Brinkmann [55] contends that diagnostic and self-help narratives medicalize normal challenges and narrow the vocabulary available for interpreting life experience. Madsen [52, 56] similarly critiques the dominance of psychological discourses in Norwegian society, which encourage self-optimization at the expense of structural or relational understandings. The high prevalence of diagnoses in our sample (36%, not reported data) reflects how psychological labels have become common interpretive tools for existential difficulty. This may risk reinforcing an individualised view of suffering, detaching it from broader social contexts.

Binder’s existential perspective deepens this interpretation. In *Suffering a Healthy* Life [57], he calls for a reintegration of suffering into the concept of well-being, challenging the diagnostic tendency to treat discomfort as pathology. The high scores for crisis of meaning in our sample echo this tension: a cultural aversion to suffering may reinforce mental health challenges by framing existential discomfort as dysfunction. Binder, like Rosa and Brinkmann, advocates for embracing struggle as part of life – thereby enabling greater resilience and more meaningful engagement.

These perspectives converge in suggesting that well-being depends not only on internal coping or professional intervention but also on social, educational, and cultural contexts that enable individuals to experience connection, resonance, and existential depth. Future research should investigate how young people can be supported in reframing suffering meaningfully and how educational institutions can foster these reflective capacities.

### Screen time, social life, gender, and mental well-being

This study’s findings add to the growing body of literature on the psychological impact of digital technologies on young people. Screen time was positively associated with poorer mental health, as indicated by higher SCL-10 scores, while community connection showed a negative correlation with symptom severity – emphasising the protective function of meaningful social relationships. Additionally, participants with high screen use reported significantly more sleep-related problems (one of the SCL-10 items), and video gaming, although often framed as a social activity, was likewise associated with higher symptom levels.

Gender and identity emerge as important factors. Female participants reported significantly higher levels of distress than males, a finding consistent with national surveys [1, 58]. This gender gap in mental health remains a paradox in egalitarian societies like Norway, where women – formally – have high levels of equality. Recent research suggests that the persistence of these disparities may be explained by intersecting pressures, including heightened academic and social expectations and exposure to body image ideals on social media [59–61]. While structural equality has advanced, these psychosocial stressors may disproportionately affect young women, contributing to their elevated symptom scores in our study. Beyond the female–male disparity, participants identifying outside the gender binary reported the highest symptom scores in our sample, with a gap substantially larger than that between females and males. Although the number of participants in this group was small (6%), the difference is consistent with international findings that non-binary youth often face elevated risks of mental distress due to minority stress, discrimination, and lack of institutional support [62]. At the same time, it is important to note that causality may point in multiple directions: while minority stress can exacerbate psychological distress, mental health challenges may also influence self-identification processes.

Beyond the well-documented association of gender with mental health symptoms, this study highlights screen time as a significant predictor of diminished meaning in life. This finding is noteworthy, as previous research has focused primarily on symptomatic outcomes of digital engagement, while largely overlooking its existential implications. Our results, though obtained by just one screening question, suggest that high screen use may not only increase distress but also weaken young people’s orientation toward meaningfulness.

Recent work by Haidt [6] underscores these concerns by linking the rise in adolescent anxiety, depression, and self-harm – especially among girls – to the proliferation of smartphones and social media platforms. He argues that in-person socialisation and exposure to real-world challenges are essential for developing psychological resilience. Without examining causality, our findings resonate with this claim: stronger community connection, less screen time, and more time in nature (so-called “green time”) correlate with better mental health. This aligns with findings in ecotherapy and environmental psychology. Oswald, Rumbold et al. [63], for example, reviewed studies showing that while screen time increases anxiety and stress, green time is associated with improved mood and lower physiological arousal. Similarly, Michaelson, King et al. [64] found that higher screen time among Canadian adolescents predicted weaker connections to nature, and qualitative interviews revealed that youth often prioritise digital engagement due to convenience, habit, or perceived safety – yet many also experienced renewed well-being after screen disconnection.

However, critiques of Haidt’s position rightly point out the difficulty of establishing causality in this field. Odgers [65] and others argue that the mental health effects of social media depend on how it is used – for example, to seek support or maintain friendships. Similarly, Kysnes, Hjetland et al. [66] suggest that using social media for emotional sharing may buffer against distress. Our study cannot determine causality, and the use of self-report measures introduces the risk of attenuation bias [67], where measurement error dampens true associations. Still, even small observed correlations may reflect meaningful relationships obscured by noisy instruments – especially when measured at the group level.

Notably, several Norwegian studies do support causal interpretations. Brunborg, Skogen et al. [68] and Brunborg and Andreas [69] found longitudinal links between problematic screen use and mental distress among youth. Nonetheless, purely causal models may be too narrow to capture the circular, systemic nature of digital technology’s influence. Mental health evolves through complex interactions between genetic, psychological, social, and cultural variables [70] – including, crucially, the meaning individuals assign to their activities. From this perspective, the findings might reflect an inverse of positive psychology’s basic presumption [71]: Instead of doing something meaningful together, social media increase the possibility of doing something meaningless alone.

In Norway, the debate around screen time has intensified. A 2023 report from the Norwegian Media Authority [72] noted that many children access social media well before the age of 13, often encountering harmful content and cyberbullying. In response, the government has proposed stricter guidelines for digital use in schools [73]. Norwegian research further confirms that face-to-face interaction is a protective factor for mental health [74], while exclusion or bullying on digital platforms is consistently associated with higher mental distress [13].

Taken together, these findings support the need for balanced digital engagement and environments that facilitate meaningful interpersonal connection. Educational settings and public health initiatives should promote digital literacy alongside relational and nature-based activities. Future research could evaluate such programs and explore contexts where screen use remains low – not only to assess outcomes but to identify what enables young people to choose more resonant, less alienating forms of connection.

### Implications

The findings of this study highlight the importance of fostering meaningful social interactions, time in nature, and opportunities for personal development to support young adults’ mental health. Schnell’s framework of meaning in life shows that meaningfulness, community, and spirituality function as protective factors. These results suggest that interventions should move beyond symptom reduction and actively strengthen opportunities for meaningful engagement.

Educational and community initiatives are particularly relevant in this regard. Norwegian folk high schools as value-based institutions [26], with their emphasis on community, reflection, and self-directed growth, represent a promising context for examining how structured environments may enhance meaning and well-being. Their pedagogical model offers young people a rare space to explore values, develop resilience, and cultivate belonging outside of competitive or performance-driven settings.

School-based programs fostering community participation (e.g., organized group activities), structured opportunities for outdoor experiences, and digital literacy curricula that encourage mindful screen use may be promising avenues for supporting youth well-being also in regular schools or higher education. While the present study cannot evaluate interventions directly, our findings appear to merit systematic evaluation. Future research should evaluate the long-term impact of such programs, ideally through longitudinal and qualitative designs, to better understand how young adults construct meaning and sustain well-being in a highly secular, digitalised society.

### Limitations and strengths

This study has several limitations that should be acknowledged. First, the reliance on self-reported measures – including the SCL-10 and screen time – introduces potential biases and measurement error, which may attenuate the observed correlations; furthermore, the exclusive reliance on the SCL-10 as a measure of mental health does not capture specific domains such as emotional regulation or positive psychological functioning. Second, the cross-sectional design precludes causal inference; while associations can be observed, the directionality of effects remains uncertain. Third, although the sample includes participants from both universities and folk high schools, it may not fully represent the broader population of Norwegian young adults, especially those not currently enrolled in education or training. To ensure anonymity and focus on existential and behavioural factors, we collected only age, gender, and migration background, and refrained from open-ended questions. This limits the examination of finer sociodemographic effects (e.g., parental education, socioeconomic status, personality traits) and subjective perspectives on constructs like meaning or purpose. Prior research shows these factors can shape both mental health and meaning in life [40], and future studies should integrate them more systematically. Moreover, the sample was culturally homogeneous, with very few participants reporting an immigrant background. This partly reflects Norwegian society but limits the transferability of findings to other cultural contexts, even within Scandinavia [2].

Despite these limitations, the study also offers important strengths. The relatively large sample size (*n* = 713) enhances statistical power and the reliability of estimates. The used instruments (SCL-10 and MAPS) ensure robust measurement of mental health and meaning-related constructs. Furthermore, the study contributes novel insights by examining a combination of behavioural and existential variables – including social commitment, screen time, and nature exposure – within a theoretically grounded framework. By integrating Schnell’s meaning theory with sociological perspectives from Rosa and the salutogenic tradition, the study contextualises individual experience within broader cultural patterns. This multidimensional approach strengthens the study’s relevance for both empirical research and policy development.

Future research should build on these findings through longitudinal designs, more diverse and inclusive samples, and the incorporation of objective or multimodal measures of digital use, social interaction, and meaningful activities as well as additional mental health measures.

## Conclusion

This study highlights the complex interrelations between mental health, meaning in life, and social behavioural factors among young adults in a highly secular Norwegian context. The findings emphasize the protective function of meaningfulness and social commitment, showing that lower levels of meaningfulness, community connection, and time spent in nature are significantly associated with poorer mental health outcomes. These results reinforce the need to support young people’s engagement in meaningful, embodied, and socially grounded activities.

At the same time, the negative associations between screen time and mental health underscore the psychological costs of excessive digital engagement. As digital technologies increasingly shape daily life, the importance of promoting a balanced digital culture and safeguarding opportunities for genuine, in-person connection becomes more urgent.

Although the cross-sectional design and reliance on self-reported measures limit causal inference, the study contributes novel evidence linking meaning and mental health within a sociocultural and existential framework. Future research should use longitudinal and mixed-method designs to examine the development and sustainability of meaningful engagement over time.

Policymakers and educators should consider interventions that expand young people’s access to diverse sources of meaning – such as nature experiences, social participation, and creative or spiritual practices – while also addressing structural barriers to well-being. By drawing on both existential and psychological perspectives, such approaches may help counter rising mental distress and foster resilience in an increasingly complex world.

## Data Availability

The data supporting the findings of this study are part of an ongoing longitudinal research project and are not publicly available due to project constraints. However, the data may be made available by the corresponding author upon reasonable request.
